# Software-Based Process Simulation and Feasibility Assessment of Black Soldier Fly Larvae Fatty Acid Extraction and Fractionation

**DOI:** 10.3390/ani12182349

**Published:** 2022-09-08

**Authors:** Felix Subakti, Chung-Hsi Chou, Misri Gozan, Yuan-Yu Lin

**Affiliations:** 1 The Master Program in Global Agriculture Technology and Genomic Sciences, International College, National Taiwan University, Taipei City 106, Taiwan; 2 Department of Chemical Engineering, Universitas Indonesia, Kampus UI Depok, Depok 16424, Indonesia; 3 Department of Animal Science and Technology, National Taiwan University, Taipei City 106, Taiwan

**Keywords:** biorefinery, black soldier fly, fatty acids, process simulations, insect products, waste management, process optimizations, industrial upscaling

## Abstract

**Simple Summary:**

Upscaling is a significant part of implementing industrial research in a large-scale market. In some cases, this means constructing a simulated factory version before establishing an in situ pilot-scale plant to predict and increase any chances of feasibility. A black soldier fly larvae fatty acid extraction factory may find its merit in the proper utilization of the increasingly explored potential of insect larvae. The factory predicted and assessed the feasibility of black soldier fly larvae under three different feed varieties. Fatty acids would be the final product, with several waste products and by-products.

**Abstract:**

Black soldier flies have been studied as an alternative animal feed. On the other hand, they could be used to yield an abundance of fatty acids. Their omnivorous diet and low space requirements allow for the mass breeding of black soldier flies, using widely available food wastes as feedstock. This study simulates the industrial upscaling of an extraction process for black soldier fly larvae using SuperPro™ 9.5 simulation software. This software contains an extensive material library that regulated physical data for the chemical composition of the larvae and the products. It also bundled several types of bioreactors utilized in bioprocessing. The scheduling of the plant was aided by SchedulePro, which allows for the generation of batch durations and Gantt charts. Four fatty acids were chosen as the main revenue source, with simulated proteins assigned as by-products of the plant. Ash and cellulose were the wastes of the plant, and were separated through multiple filters. The plants were later assessed for their economic feasibility. The kitchen waste plant was the most profitable, and the control variable was the only unprofitable plant. These results may have been impacted by the waste content found in the control variable and the abundance of revenue products in the kitchen-waste-fed larvae.

## 1. Introduction

In recent years, the utilization of deliberately bred insects has piqued the interest of the academic world. Insects have been investigated thoroughly for their benefits in medicinal applications, animal feed, and even pest control in commercial agriculture. One insect, in particular, has attracted the interest of researchers—the black soldier fly. This highly adaptable [1], fast-breeding flying insect produces docile soft-shelled larvae that are rich in proteins and fats. These larvae have been researched extensively for their content of fatty acids [2].

Besides proteins and fatty acids, black soldier fly larvae also produce ash and fiber wastes during their metabolism [3]. The contents of a black soldier fly larva are mainly affected by its feedstock during the maturation period [4].

In recent years, black soldier fly larvae have been used for the small-scale waste treatment of organic materials such as rice straw, fishery wastes, fecal sludge, and industrial food wastes [5]. The demand for black soldier fly larvae has also been pushed upwards due to their use as a feedstock for carnivorous land-farmed fish commodities, as they were deemed an ideal substitution for more climate-sensitive insect-based feed, such as feed made from crickets and grasshoppers [6].

The fatty acids found in black soldier fly larvae have known benefits in medicine, food, oleo chemistry, and biochemistry, with an increasing demand locally in Taiwan and globally [7,8]. In Mediterranean Europe, these lipids can be found in olive oil [9]; in tropical Africa and Southeast Asia, oleochemicals are harvested from palm fruit bunches [10]. Taiwan, having no such biodiversity advantage compared to the other two regions, could achieve self-reliance using the lipids found in black soldier fly larvae. Mass production in a plant system would be mandatory [11] to fully utilize black soldier fly larvae as a source of lipids. The plant would handle the extraction process of fats from the larvae, separating them into each component and packaging them for distribution [12]. Before physically establishing a larva-harvesting plant, a software-based simulation could be constructed to test the feasibility of such an endeavor. A computerized simulation was chosen, as it could reduce potential mistakes, predict hurdles that may occur, and reduce the cost required to test the plant in situ [13].

Establishing a plant usually requires a concise schematic of the unit processes involved in the production, from its feedstock to the commercial product [14]. However, as black soldier fly larvae are an uncommon feedstock for oleochemicals, they have pushed forward the feasibility issue. Several issues were posed when constructing a plant for said oleochemical products, such as inseparable impurities and expensive units [15], which drove up the capital starting costs and the number of inaccurate calculations within the software. This research aimed to construct a black soldier fly larva fatty acid plant using simulation software and analyses throughout the entire process. The simulation produced a scheme feasible from both an engineering and economic perspective. An industrial mode of production for black soldier fly larvae fatty acids was expected to grant higher economic value. This valorization allowed for more sustainable fatty acid production in regions where such an endeavor was previously impossible due to a lack of conventional plant-based sources.

## 2. Materials and Methods

The simulation was conducted using SuperPro™ 9.5 (Intelligent, academic license). It was used with cooperation with Misri Gozan from University of Indonesia in Indonesia as the academic license owner. The tools were taken from parts of the software library and sized accordingly.

### 2.1. Mode of Operation

The plant was simulated under a long-term batch system. The operation time was set to 7920 h annually. The deliberate choice of operating time was to account for some downtime that occasionally occurs for upgrades or equipment maintenance.

### 2.2. Definition of Components in the Simulation

The components required for this simulation to run are presented in Table 1. The components were collected in the SuperPro™ built-in database; in this case, several additional components were inputted into the library due to default collection limitations.

### 2.3. Fly Larvae Modelling

The input materials of the plant—in this case, fly larvae—had multiple compositions programmed through the customization of the SuperPro™ library and presented as a summary in Table 2. The baseline ratios were procured through a literature review. The factory did not include the waste treatment facility that would be the breeding site of the fly larvae. It was also assumed that the fly larvae breeding would incur little to no cost, thus reducing the required capital for procuring the main feed.

### 2.4. Alcalase Enzyme

The alcalase enzyme is a protease enzyme that enables the induction of proteolysis. In the context of proteases, alcalase is considered a “serine endopeptidase”, which provides information about the catalytic structure of the enzyme, which is known for its classical catalytic triad of amino acids, with serine being one of them [15]. This enzyme also cleaves proteins in the middle of the amino acid chain [16]. The ideal conditions for this enzyme to function as a catalyst are a pH of 9.5 and a temperature of 56 °C [17].

The alcalase enzyme’s effect on black soldier fly larvae was assessed by Assis et al. [18] on a laboratory scale. The enzyme treatment was performed on a larvae cream slurry and was compared to blanched, washed, and sonicated treatments and observed for cream fractionation after a centrifugation treatment. In that paper, enzyme introduction was recommended to encourage the hydrolyses of the proteins that comprised the fly larvae tissues. This allowed for a more thorough extraction of the lipids.

### 2.5. Unit Processes Involved in the Plant

The unit processes performed here followed the unit library provided by the SuperPro™ simulator software. The labelling followed the default automated unit labelling system built into the software; the final product may differ in labelling, but the overall functions typically remained the same. The details regarding the unit processes can be read in Table 3.

The whole factory can be split into three main sections [19]: the mechanical pre-treatment, where the larvae will be washed and ground into basic sludge; the reactor process, where the sludge will be incubated with an alcalase enzyme; and finally, the separation process, which includes centrifugation, waste separation, and distillation.

### 2.6. Process Parameters

Each unit within the simulation was customized according to the throughput and composition of each step of the batch’s input stream. The software automated the labelling of each unit with built-in numberings. The two most common parameters in all units were temperature and pressure. The factory did not operate under a vacuum, and the air was considered to be a mixture within the simulation library. The details regarding the processes utilized are available in Table 4.

### 2.7. Contents of the Flow

The plant parts consisted of the flow of raw material and intermediary substances in multiple forms. The expectations regarding what should be found in a certain flow for the whole simulation are listed in Table 5. However, due to the process’s environmental parameters affecting the physical properties of certain components, mixed with equipment sizing and limitations, some slight discrepancies in the flow content were expected.

The waste streams were split into wastes 1 and wastes 2 from the order of placement within the factory. Wastes 1 assigned towards the first filtration unit and waste 2 assigned to second filtration unit. The Reusable Water Streams were split in the same manner, focusing on centrifugation unit and distillation tower respectively. Streams labelled as Impure products were vaporized fatty acids needing condensation to be in a packageable form

The calculated contents of the flow were recorded similarly to Table 5, with an additional column that shows the calculated contents that diverged from the expected contents. The “purpose” part of the table column states the function of the substance in the whole grand scheme of the production line. The flow labels are a combination of default software-assisted labelling and user-inputted labelling.

### 2.8. Plant Consumables

Plant consumables are any material inputs that impact the process by manipulating environmental parameters instead of being involved in the processes themselves [20]. These consumables were recorded in the plant in Table 6 with their suitable quantitative unit, preferably in SI units, and their respective functions. These consumables also directly impacted the operational costs, and were thus grouped alongside those costs during the economic assessments.

## 3. Results

### 3.1. The Plant Schematics

The authors constructed a plant process flow using SuperPro™ simulation software. The plant itself contained four major revenue flows and three major waste flows. The revenue flows consisted of lauric acid, stearic acid, myristic acid, and palmitic acid. The ash waste and cellulose waste outputs comprised the main waste products of the facility, along with a freeze-drying exhaust waste output. A secondary revenue was established on the protein output flow, which utilized the default protein properties provided by the built-in library. 

**Figure 1:**
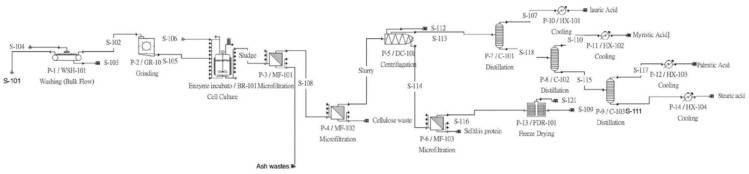
The factory schematics.

Figure 1 gave an overview of the process simulation as generated by SuperPro™ 9.5. The simulation contains a washer, a grinder, a reactor, three filtration units, a centrifuge, three distillation towers, four heat exchanger units and one freeze drying unit.

### 3.2. Time Constraints of the Factory

#### 3.2.1. Plant Operational Scheduling

The Plant was operated under a pseudo-continuous mode of operation, allowing for an active period of 330 days as shown in Table 7. The discrepancy towards the standard 365 or 360 days could be meant to properly represent maintenance and upgrade downtime often occurring in larger-scale biorefineries like this one. A single batch of fly larvae could be properly conducted for a quarter above 7 hours, easing the scheduling burden of future project managers. Most labor works were done using workdays consisting of 8 active hours. Table 7 also specified a recipe cycle time between batches.

#### 3.2.2. Plant Batch Scheduling

**Figure 2:**
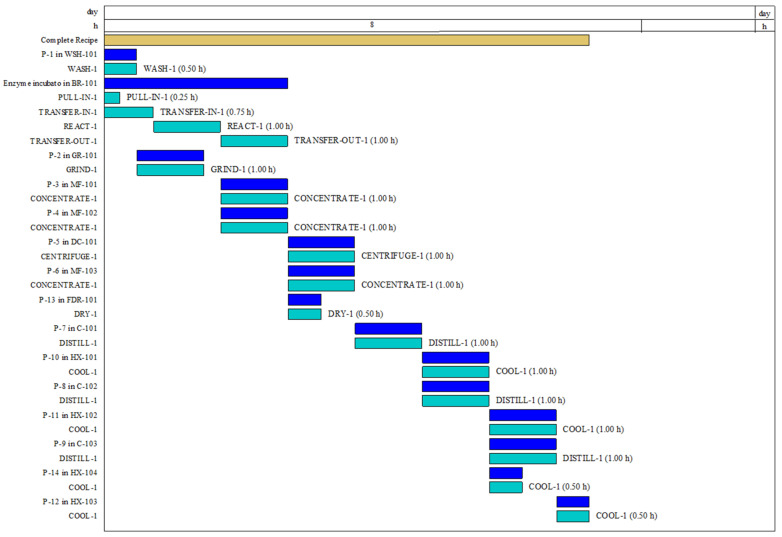
The production plant Gantt chart for scheduling purposes.

The plant batch was conducted using several inter-dependent processes within the plant. These interdependent processes often used the output of a prior process as the input of its succeeding process. Some processes also worked in parallel, which means the process was semi-continuous, with the output moving into the subsequent processing unit. Most of the processes shown in the Gantt chart were set within the constraints of the software as seen in Figure 2.

Many processes were set to one hour to suit a typical workday of 8 h, with the data visualization in Figure 2 were set in 8 h cycles. The pull-in and transfer-in processes of the factory were set to less than an hour to reasonably scale with the main process. This was performed in contrast to the default flow or process setting, which followed a throughput-based calculation.

### 3.3. The Material Balance Report

The material balance report can be broken down into the summary and the stream details [21]. The summary as presented in Table 8 was split into 3 columns, consisted of the alcalase enzyme, the fly larvae, and the water input. The water input was passively calculated through a combination of the per-unit input, adjusted by the researcher, and the calculated demand of some unit processes that were not within the researcher’s control. The water demand also counted in more obscure process demands, such as a thermal jacket with fluid convection.

### 3.4. The Revenue Classification by Type of Fatty Acid

**Figure 3:**
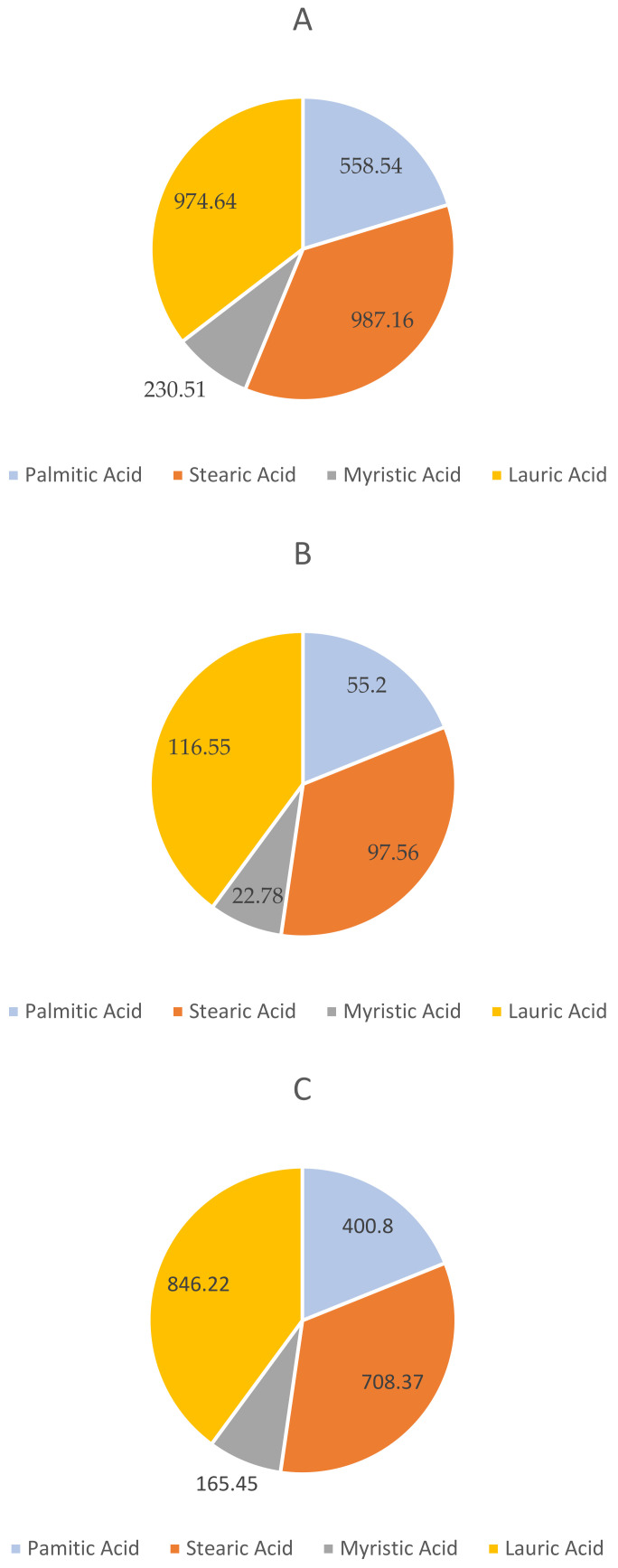
The revenue material flow (in kg) for (**A**) kitchen waste; (**B**) control; and (**C**) spent grain factory simulations.

The pie charts that comprised Figure 3A–C show the four major fatty acids, which were split within the revenue stream under observation within the simulation. Lauric acid, stearic acid, palmitic acid, and myristic acid were quantified by their mass in their liquid form post-condensation.

## 4. Discussion

### 4.1. Materials and Energy Balance

The generated income was from the lauric acid, stearic acid, palmitic acid, and myristic acid [22]. These four revenue streams were influenced heavily by the feedstock of choice for the larvae. Most of these outputs can be found from fly larvae fed with kitchen wastes, with spent-grain larvae found to yield the lowest revenue. It was decisively found that the waste products were found in higher amounts when the spent-grain larvae were used as the input into the plant. This result aligned well with the baseline composition previously established through the literature review.

The waste product was programmed to be a cost-inducing component, as the plant did not possess any waste treatment installation. A possible third-party entity handled any form of waste. This is in stark contrast to some factories that center their profitability on a built-in waste treatment system, which eliminates the need for a third-party waste treatment facility [23]. Thus, waste treatment-related costs were the highest within the spent-grain plant.

The fiber-rich spent-grain fly larvae diet may have caused a reduction in fatty acid revenue components. Due to an inverse ratio between the chemical makeup of fly larvae, the presence of fatty acids and fiber is competitive. This contrasts kitchen-waste-fed larvae or animal-waste-based feed for the larvae [24].

The authors deemed the two filters installed within the factory necessary, yet they could pose issues. Both the waste separation outputs contained fatty acids, which may have reduced the potential profits, as some of the revenue material was removed as waste by the passive indiscrete filter membrane.

### 4.2. The Economic Breakdown

The fatty acids were programmed with a fixed amount of costs, so the researchers deemed the price changes to be unimpactful to the overall profitability of the factory. It was also set under the assumption that animal-based fatty acids would not experience any issues becoming accepted within the Taiwanese market.

The cost within the factory was hiked immensely by the distillation segment, owing to the multiple towers required to separate the purified forms of the fatty acids, all of which also required a significant energy input to operate. This was surprisingly in line with previous fractionation tower research [25]. Following suit was the bioreactor vessel that consumed the alcalase enzymes, which in this factory was industrial-grade and was used once per batch.

The factory payment system can be assessed using a cash flow analysis, and in that case, the modelling followed a 20-year payment period. In most cases, a factory will start off as unprofitable before returning the capital after at least 10 years. The economics were based on a 6% flat tax rate. Factory profitability can be measured by assessing IRR and NPV [26]. The internal rate of return (IRR) is the discount rate that makes the net present value (NPV) of a project zero [27]. Meanwhile, the net present value (NPV) is defined as the value of all future cash flows (positive and negative) over the entire life of an investment discounted to the present [28].

It can be seen from Table 9 that the kitchen-waste factory showed the highest IRR of the three factories, indicating a great rate of return on the investment capital. Meanwhile, the worst between the three was shown for the control variable, which showed no profitability whatsoever.

### 4.3. Possible Future Improvements

Several waste products could be addressed better, particularly cellulose and ash, which both have established methods of waste treatment. Implementing a more discrete membrane for the filter may also be beneficial for reducing the potential wastage from carried fatty acids, which reduced profitability. In addition, the alcalase enzyme could be reused with a limited lifespan if the enzyme was introduced in an immobilized or encapsulated form.

## 5. Conclusions

The factory simulation deemed the extraction and refining of fly larvae fatty acids to be feasible through engineering and economical viewpoints. There are certain considerations to be made regarding the waste treatment of the plant, as cellulose-based wastes may add a lot of additional costs to the factory’s capital requirements.

## Figures and Tables

**Table 1:** The components of the simulation.

Components	Definition
Fly larvae	A mixture of water, proteins, cellulose, crude oils, and ash wastes
Crude oils	A mixture between lauric acid, stearic acid, and myristic acid in their respective ratios
Proteins	Available in SuperPro™ database
Alcalase enzyme	Inputted by the author
Lauric acid	Inputted by the author, with a boiling point of 298.8 °C
Myristic acid	Inputted by the author, with a boiling point of 326.2 °C
Palmitic acid	Inputted by the author, with a boiling point of 348 °C
Stearic acid	Available in SuperPro™ database
Cellulose	Available in SuperPro™ database
Ash	Available in SuperPro™ database
Water	Available in SuperPro™ database
Air	Available in SuperPro™ database

**Table 2:** Chemical composition of black soldier fly larvae, with percentages adjusted [4].

Parameters	Chicken Manure	Kitchen Waste	Spent Grain	Control
Dry matter	80.7 ± 1.2	87.7 ± 1.0	83.1 ± 1.6	32.7 ± 5
Ash	9.3 ± 1.8	9.6 ± 1.6	11.6 ± 0.5	15.9 ± 3.1
Organic matter	59.8 ± 0.4	90.4 ± 1.6	88.4 ± 0.5	84 ± 1.4
Crude protein	41.1 ± 0.3	33.0 ± 1.0	41.3 ± 0.5	44.7 ± 3.4
Neutral detergent fiber	21.9 ± 0.6	20.4 ± 0.6	28.6 ± 1.0	10.6 ± 4.2
Acid detergent fiber	12.6 ± 0.3	13.2 ± 0.1	15.0 ± 0.8	19 ± 2.2
Ether extract	30.1 ± 0.4	34.3 ± 0.4	31 ± 0.4	9.7 ± 3.3

**Table 3:** The unit processes involved in the plant.

Unit Type	Unit Label	Function in the Process
Cold water washer	WSH-101	Washing harvested black soldier fly larvae
Bioreactor incubation unit	BR-101	Inducing a hydrolysis reaction on the larvae
Electric grinder	GR-101	Reducing the molecule size of the larvae suspension mechanically
Centrifugation unit	DC-101	Separating fluid in the mix by density
Membrane-based microfiltration	MF-101	Filtering cellulose from the substrate
Membrane-based microfiltration	MF-102	Filtering out ash waste from the substrate
Freeze dryer	FDR-101	Removing water from the protein by-products
Land-based expedition	P-11	Pointing at the bulk transportation and selling of the protein by-products
Ultrafiltration membrane unit	UF-101	Filtering cellulose from reusable water
Fractional distillation tower (1)	P-1/C-101	Separating lauric acid and water from the crude oil mix
Fractional distillation tower (2)	P-2/C-102	Separating stearic acid from myristic acid
Backwash adsorption column	GAC-101	Purifying lauric acid from water contents
Heat exchanger 1 (cooling)	HX-101	Condensing lauric acid vapor into a packageable liquid form
Heat exchanger 3 (cooling)	HX-103	Condensing stearic acid vapor into a packageable liquid form
Heat exchanger 4 (cooling)	HX-104	Condensing myristic acid vapor into a packageable liquid form
Heat exchanger 2 (cooling)	HX-102	Condensing palmitic acid vapor into a packageable liquid form

**Table 4:** Process conditions within the plant.

Operations	Unit Label	Temperature (°C)	Pressure	Conditions
Larvae washing	WSH-101	Room temperature	Atmospheric	Removal of 66% ash and 38% cellulose
Incubator	BR-101	60	Atmospheric	
Mechanical molecule size reduction	GR-101	Room temperature	Atmospheric	Calculated throughput of 9174 kg/h
Fractional centrifugation	DC-101	Room temperature		Minimum diameter of 20 microns; 900 kg/m^3^ density
Cellulose removal filtration	MF-101	Room temperature	10.132 bar (automated)	Rejection rate of 0.999 for cellulose, 5% denaturation, 1100 g/L maximum density, and user-inputted flux of 20 L/m^2^ h
Ash removal filtration	MF-102	Room temperature	10.132 bar (automated)	Rejection rate of 0.999 for ash, 5% denaturation,1100 g/L maximum density, and user-inputted flux of 20 L/m^2^ h
Protein freeze-drying	FDR-101	12	1.013 Bar	Sublimation rate of 10 mm/h
Domestic transport	P-11	Not applicable	Atmospheric	A total of 20 tonnes per shipment
Reusable water filtration	UF-101	Room temperature	10.133 bar (automated)	Rejection rate of 0.999 for cellulose, 5% denaturation, 1200 g/L maximum density, and user-inputted flux of 20 L/m^2^ h
Lauric acid fractionation	P-1/C-101	297.7	1.013 Bar	Reflux ratio of 14.404; 22 stages
Myristic acid fractionation	P-2/C-102	326.2	1.013 Bar	Reflux ratio of 1.186; 43 stages
Palmitic acid fractionation	P-3/C-103	348.5.2	1.013 Bar	Reflux ratio of 0.984; 43 stages
Condenser exchanger	HX-101	80	2	Flow of 5.54 MT/h
Condenser exchanger	HX-102	80	2	Flow of 1.1 MT/h
Condenser exchanger	HX-103	80	2	Flow of 32.99 MT/h
Condenser exchanger	HX-104	80	2	Flow of 65.2 MT/h

**Table 5:** The contents of active flows within the plant.

Low Label	Expected Contents	Purpose of the Flow
Larvae feed	Larvae feed	Feed input
Wash water	Cool water	Required input for the washer
Wastes 1	Ash, cellulose, and water	Waste stream
Crude suspension	Broken-down contents of the black soldier fly larvae	Process flow; feed for the incubator
S-102	Alcalase and water	Input feed flow for the incubator
S-104	Larvae contents	Process flow; feed for the grinder
Sludge	Larvae contents, water, and residual enzymes	Process flow; feed for the filter
S-108	Crude oils, water, proteins, residual enzymes, and ash	Process flow; feed for filter 2
Cellulose waste	Cellulose and water	Waste output flow
Ash waste	Ash and water	Waste output flow
Slurry	Residual enzymes, crude oil, protein, and water	Process flow; input for the centrifuge
Oil feed	Crude oil mix	Process flow; input for the fractionation tower
S-112	Cellulose, residual water, and water	Process flow; input for ultrafiltration unit
Wet protein meals	Proteins, water, and residual enzymes	Process flow; input for freeze-drying
Reusable water 1	Water	Non-revenue output flow
Wastes 2	Cellulose and trace amounts of water	Waste output flow
S-103	Stearic acid (liquid) and myristic acid (liquid)	Process flow; input for distillation tower
S-110	Lauric acid (liquid) and water (liquid)	Process flow; to be water-adsorbed
S-105	Myristic acid (vapor)	Process flow; to be cooled to a liquid
S-111	Stearic acid (vapor)	Process flow; to be cooled to a liquid
Impure product 1	Lauric acid (vapor)	Process flow; to be cooled to a liquid
Myristic acid product	Myristic acid (liquid)	Revenue output flow
Stearic acid product	Stearic acid (liquid)	Revenue output flow
Lauric acid product	Lauric acid (liquid)	Revenue output flow
Backwash water	Water	Input flow for adsorption tower
Reusable water 2	Water	Non-revenue output flow

**Table 6:** The consumables within a single plant.

Consumable	Main Dimension	Main Measuring Unit	Function	Source
Superheated steam	Weight	Metric tonnes (MT)	Heat transfer agent	SuperPro™ base library
Cooling water	Weight	Cubic meters (m^3^)	Heat transfer agent	SuperPro™ base library
Electricity	Energy	KiloWatt/hour (kWh)	Unit power supply	Superpro™ base library
Superheated steam	Weight	Metric tonnes (MT)	Heat transfer agent	Inputted by the author
Dead-end filtration membrane	Area	Square meters	Separation medium	SuperPro™ base library
Ultrafiltration membrane	Area	Square meters	Separation medium	SuperPro™ base library
Fractionation packings	Spatial density		Fractionation heat spread	SuperPro™ base library

**Table 7:** The overall factory process assessment.

Annual Operating Time	7919.00
Recipe Batch Time	7.25
Recipe Cycle Time	2.75
Number of Batches per Year	2878.00

**Table 8:** Bulk material consumption for each batch.

Material	kg/yr	kg/batch
Alcalase enzyme	287,800	100.00
Fly larvae	28,780,000	10,000.00
Water	103,775,015	36,058.03
Total	132,842,815	46,158.03

**Table 9:** The IRR and tax summary of the three factories—kitchen waste (A), spent grain (B), and control (C).

IRR/NPV SUMMARY—A					
IRR Before Taxes	7.73%	Interest %	7.00	9.00	11.00
IRR After Taxes	5.23%	NPV	−2638.00	−5126.00	−7120.00
**IRR/NPV SUMMARY—B**					
IRR Before Taxes	7.73%	Interest %	7.00	9.00	11.00
IRR After Taxes	5.23%	NPV	−2638.00	−5126.00	−7120.00
**IRR/NPV SUMMARY—C**					
IRR Before Taxes	−100.00%	Interest %	7.00	9.00	11.00
IRR After Taxes	−100.00%	NPV	−110,454	−99,792	−90,788

## Data Availability

Data available by contacting Felix Subakti or Yuan-Yu Lin.
